# Somnoform; Its Physiological Action and Its Administration

**Published:** 1905-01-15

**Authors:** Florestan Aguilar

**Affiliations:** Madrid, Spain


### SOMNOFORM: ITS PHYSIOLOGICAL ACTION AND ITS ADMINISTRATION.

BY DR. FLORESTAN AGUILAR, MADRID, SPAIN.

Everything tending to the alleviation of pain is of such importance to those devoted to the practice of dentistry that we observe with a marked degree of interest anything pertaining to the fields of general and local anesthesia.

The discovery of Dr. Rolland, director of the Dental School of Bordeaux, of the mixture which he has named *somnoform*, and which consists of ethyl chlorid 60 percent, methyl chlorid 35 percent, and ethyl bromid 5 percent, I consider to be one of the most important clinical discoveries of modern times. At the congress of the Association Francaise pour l'Avancement des Sciences held in 1901, Dr. Rolland presented for the first time an essay on somnoform, accompanied by clinical demonstrations. Since then its use has been largely generalized in Europe, where over 25,000 patients have been anesthetized by Dr. Rolland himself. These cases, together with others recorded at the hospitals of England and France, and in the medical department of the University of Madrid, constitute a total of over 100,000 cases in which the properties of this anesthetic have been tried.

A series of 100,000 favorable cases confirms without doubt the satisfactory laboratory experiments previously carried out, showing conclusively the advantages of this agent over chloroform and nitrous oxid, from the minimum of danger incurred by its administration when employed for brief anesthesia.

Upon the brilliant studies undertaken by Dr. Rolland, and the writer's own experience and investigations in the laboratory of physiology of the University of Madrid, the notes have been prepared which I now have the honor of submitting to your consideration and discussion.

In order that an anesthetic should enter the respiratory tract and act on the nerve centers, it most be in the gaseous form; and the rapidity of its absorption is in direct ratio to its degree of diffusibility. This is the force which causes the blood corpuscles to become saturated with the narcotic vapors instead of with oxygen; therefore the action of the gas on the nervous system will be rapid in proportion to the rapidity of saturation. Dr. Rolland presents the problem of anesthesia in the following propositions:

First: To produce anesthesia it is necessary that the tension of the anesthetic gas be superior to that of oxygen, so that it may in a certain proportion, take the place of the latter in the pulmonary alveoli.

Second: The tension of a gas being proportionate to its volatility, the more volatile the gas is, the easier can it be made to take the place of oxygen.

Third: The ideal anesthetic, if such be attainable, would be the one behaving in its conditions of entry, of sojourn, and of exit from the body, as does oxygen.

If we follow the course of oxygen in the body, we see that the red blood corpuscles, after becoming charged with oxygen in the lungs during inhalation, distribute it to the tissue throughout the body. The blood corpuscles have their period of activity during their course through the arterial system. When the oxygen has been given up, the corpuscles return through the venous system to the lungs in an inert and dormant state; and there by contact with oxygen resume again their former activity. Now, as about twenty-five or thirty seconds are necessary for a red corpuscle after leaving the heart to return to it, we can say that in this diagrammatic division of the circulation in two parts, one arterial and the other venous, the action of the oxygen would last from twelve to fifteen seconds; therefore an anesthetic capable of being absorbed practically in the same manner as oxygen should produce its effect in about fifteen seconds, and when the administration is discontinued it should be eliminated in proportion, as the corpuscles of the blood come again into contact with the oxygen. This, almost to exact precision, is what takes place with somnoform. In the study of this physiological action we observe that somnoform produces the following phenomena.

### ON CIRCULATION.

Somnoform has a powerful action on the sympathetic system, increasing the arterial tension and the frequency of the cardiac contractions. A series of curves of the blood tension taken with the sphygmograph of Marey and the sphygmomanometer of Potain on the radial artery of Dr. Rolland showed in twenty minutes a variation of from $13\frac{1}{2}$ of normal blood pressure to $14\frac{1}{2}$, 17, 17, 13, 14, 15, 14, 14, $13\frac{1}{2}$, during, through, and after the anesthesia. The pulse which formerly was 76 per minute, presented in the same observation a frequency of 76, 84, 76, 68, 68. Respiration, which when normal was 16 per minute, went up to 28, 20, 19, 20, 20. And a careful microscopical study of the blood of subjects under somnoform showed that the anesthesia of from five to eighteen minutes' duration produced no important modifications in the blood. The urine of the anesthetized persons also remained normal.

### CLINICAL STUDY.

Somnoform, as is the case with any other anesthetic, determines in the paitent three well-defined states: First, pre-anesthetic period, or that of induction. Second, anesthetic period, or that of resolution. Third, post-anesthetic period, or that of elimination or return to consciousness.

In each one of these periods we observe two types of phenomena, subjective and objective.

The subjective phenomena in the first period are emotional: a feeling of anxiety, of suffocation, blurred vision, tinnitus aurium, light tickling in the extremities, and the strange sensation of having a warm compress on the cerebrum from the occipital to the frontal lobes.

In the second period, or that of true anesthesia, the patient experiences no sensation whatever.

The third or post-anesthetic period commences by the sensation of far-away buzzing, the reappearance of the sense of hearing, dreams of different types, gay, religious, amorous, professional, etc., generally in relation to the subject of which the patient was thinking immediately before the anesthesia. At first he fails to recognize the place and the persons that surround him, this state being followed by return of motion, with a tickling in the extremities.

*Clinical classing of cases.* Clinically we can group the patients into three classes as follows:

The first class—embracing 90 percent of all cases—is constituted of those patients who are tranquil and unresisting. With a dose of from 3 to 5 cc, in from fifteen to thirty seconds they are anesthetized, and they remain so for from fifty to seventy seconds and sometimes for nearly two minutes. When they regain consciousness they are pleased, and express satisfaction and wonder at the slight amount of inconvenience they have experienced.

The second class will be more difficult to anesthetize. It comprises the restless class of patients who involuntarily resist anesthesia. When the administration begins they fight to get the mask off the face; they swallow but do not breathe, at first; sometimes they cry out, but finally lapse into unconsciousness. These patients are found in the proportion of 8 or 9 percent.

The third class of patients is constituted of the alcoholic, epileptic, hysterical, and tobacco users. They are difficult to anesthetize, and the elimination of the anesthetic takes place slowly. They are irritable and the anesthetic seems to have on them a hysterogenic action, provoking a nervous crisis. Fortuately, patients of this kind will be found only in a proportion of about 1 percent.

### METHOD OF ADMINISTERING SOMNOFORM.

Always employ a mask or inhaler in preference to a handkerchief or waterproof cone, with which it was originally applied. The inhaler will permit, not only the exact measurement of the dose employed, but also rapid induction by reason of the total exclusion of air—a factor of great importance. After seating the patient with the head in line with the body, explain that deep inhalations should be taken, that the liquid has a slightly irritating odor, and that it will produce a quiet and agreeable sleep, particularly if he can induce himself to think of something pleasant. The pneumatic pad of the inhaler having been inflated and tried on the patient's face, pour the somnoform from the bottle into the chamber of the inhaler in the dose of 5 cc, or the contents of a capsule; close the chamber very rapidly, and instantly apply the face-piece. Generally in about twenty seconds the action of the agent will begin and the signs of general anesthesia will be evident—namely, cessation of the ocular movements, drooping of the eyelids, dilatation of the pupils, complete relaxation, occasionally rigidity of the arms, and loss of corneal reflex. The induction is complete in from thirty to forty-five seconds, and the anesthesia lasts from sixty to ninety seconds. The pulse slightly increases in frequency and tension, and the color of the face remains completely normal without trace of the cyanosis that appears when nitrous oxid is employed. When recovery of the patient begins, analgesia persists during some seconds, allowing a little more time to operate with the patient in a semi-conscious state. In four or five minutes the patient completely recovers.

In conclusion, I consider somnoform the most valuable general anesthetic in dental practice, from the rapidity of its induction (thiry seconds), and the length of available anesthesia (fifty seconds), from the possibility of administering it to all patients and without special preparation, from its pleasant effects, and from its safety, demonstrated not only by the investigations on its actions on the nerve centers, but also by a clean record of over 100,000 cases.

I may add, gentlemen, that I have here the mask that I have been employing in my practice, and also another mask that, since my arrival in America, I have found is manufactured in this country. As soon as the clinics are organized, to-morrow or the next day, I intend to give a clinical demonstration.

I have brought a patient here this afternoon whom, if the president will allow me, I will anesthetize, so that you can see how quietly the effects of somnoform are produced, and the rapidity with which anesthesia is induced by it. I have used it many hundreds of times in the clinic of the dental department of the University of Madrid. As I say in my paper, Dr. Rolland has a record of over 25,000 cases operated upon by him, and there are additional hospital records in France, England and Spain, besides private cases, which sum up over 100,000 in all.

The manufacturers prepare somnoform either in little glass capsules like this one which I have here, or in large flasks. These little capsules are convenient, because each contains an exact amount and there is no danger of administering too large an amount. The face-piece contains a piece of cotton which absorbs the required amount of somnoform. This can be changed every time a patient is operated on. The pipe is very smooth, and can be washed and made aseptic.

Now, the way in which I proceed in my practice is as stated in my paper, first to explain to the patient that the liquid has a slight pungent odor, that he should be quiet and confident and should think of something pleasant. I have observed that if at the moment one applies the face-piece reference is made to music, as, "Do you not think you are hearing some nice music; do you not think it is beautiful?" it overcomes the repugnance of the patient, and when he recovers consciousness he has recollections of a musical dream.

Dr. Aguilar then administered the anesthetic to one of the persons present. The total time taken up by the operation was announced as having been three minutes, and twenty seconds; the time required to produce the anesthesia, twenty-nine seconds; the duration eighty seconds, while the period of partial consciousness lasted to between eighty and ninety seconds.

### DISCUSSION.

The President: Gentlemen, you have heard this very interesting paper and have witnessed the demonstration by Dr. Aguilar. It is a method that has not been used, I believe, in this country so much as in France, England, Spain, and Italy. My observation while in Europe last year was that it was quite commonly administered. In the Philadelphia Hospital it is being used to a certain extent. The paper is now open for discussion.

Dr. SUBIRANA says that he can speak from the view-point of both patient and operator. While at Bordeaux he had occasion to be anesthetized by Dr. Rolland; and while under the influence of somnoform had his hands pierced with a pin and felt absolutely nothing. He was afterward operated on, and he declared himself entirely satisfied. As an operator in Madrid he has recently used somnoform very frequently in his practice with the best results. He insists on the importance of the proper technique in administration. He states that at first he had some failures but was convinced that they occurred because air was permitted to mix with the anesthetic vapor. He has also used it in connection with chloroform in general surgery, and cites a case which occurred not many days ago where he assisted a surgeon at a hospital, giving at first somnoform and finishing with chloroform, in order to prevent the period of excitement produced by chloroform. By thus commencing with somnoform he obtained much more rapid anesthesia. He says he got one of the physicians to administer somnoform to him. The physician undoubtedly gave him an overdose, and he says that he was dizzy and sick. He thinks this was due exclusively to the excess of somnoform administered to him and to the imperfection of the administration.

DR LOSADA: I may add something to what has been said in the discussion of somnoform. I myself have been anesthetized with somnoform half a dozen times at the very least. I find the action of somnoform very similar to that of nitrous oxid; these two are very much alike in their effects. I find this advantage in somnoform, that the technique is much simpler, because you do not have to carry a big gas outfit. As Dr. Subirana says, if you take an overdose of somnoform—and I have been given an overdose twice, I believe—you do not feel at all well afterward; just as when you take an overdose of gas you do not feel at all well.

Dr. Aguilar has had more practice in the use of somnoform than I have, but my experience has led me to believe that somnoform does not seem to act well on some people. In some cases there is not always a complete loss of consciousness. There is partial anestheisia, but still the patient has some idea that he is alive. In spite of this fact, however, I believe somnoform to be a very useful anesthetic for extractions and minor oral operations. The trouble with all these general anesthetics for me is that their action is very rapid, and sometimes when you have to extract two or more roots it does not give you the time necessary to carry out the operation. That is why in most of my cases I prefer local anesthesia, which does not frighten the patient at least never so much as general anesthesia, the effects of which are usually disagreeable.

A MEMBER: It seems to me that this anesthetic has some advantages over gas. The period of anesthesia is a little longer, and there is the advantage of not having a bulky apparatus; and then, too, it appeared to have an analgesic effect after the profound anesthesia had passed. I wanted to ask Dr. Aguilar if it was practicable to use it to obtund sensitive dentin in order to prepare a cavity without causing too severe pain.

DR GRIFFITHS: I would like to ask Dr. Aguilar whether it would be safe in cases of difficult extraction of roots, when the operation cannot be completed before consciousness returns, to re-administer somnoform at the same sitting.

DR. MATTHEWS: I should like to ask Dr. Aguilar to state the external symptoms of somnoform anesthesia, the effect on the pupil, on respiration, and on the pulse.

DR LOSADA: They are the same as in any kind of anesthesia.

DR. AGUILAR: (closing the discussion). In answer to the remarks made by Dr. Subirana I can only say that I agree with him as to the importance of properly administering the anesthetic. It is very important to exclude all air when once the inhalation has begun. If a perfect occlusion of the apparatus with the face be not made, a very imperfect anesthesia will result and a much larger amount comparatively of the anesthetic, and a longer time, will be required to produce unconsciousness. Therefore, with Dr. Subirana, I likewise insist upon the importance of a total exclusion of air during the administration of the gas. I may also say that it is true, as he has stated, that you can produce a very agreeable chloroform or ether anesthesia by beginning with somnoform, overcoming in this way the excitement period of ether or chloroform anesthesia.

In regard to the remarks made by Dr. Losada, I may say that I have not found that some patients cannot be anesthetized with somnoform. If you will recollect, I said in my paper that there are alcoholic and epileptics who manifest a resistance to the action of somnoform, and upon whom it produces an hysterical effect. That is, it provokes a nervous crisis, just the same as in the case of chloroform or nitrous oxid. You have all doubtless found in the administration of nitrous oxid that some patients exhibit a state of violent excitement. Happily I find the proportion of that class of patients in my practice to be only about one percent. I have made these computations after consultation with Dr. Rolland, whom I saw before I came here. He also stated that in his experience, covering 25,000 administrations only, about one percent of the patients are very difficult to anesthetize with somnoform.

Dr. Griffiths has asked if it be possible to re-administer the anesthetic; that is to say, in the case of several extractions, if the patient returns to consciousness before the operation has been completed. I have done that on several occasions without any bad after-effects.

In regard to the question as to which were the cases in which I considered somnoform anesthesia contra-indicated, I may say, in very marked valvular disorder only.

I may also say that one of the best advantages of somnoform is that it produces absolutely no change in the appearance of the face, which remains perfectly normal.—*Cosmos.*

